# The Functional Relevance of Diffusion Tensor Imaging in Patients with Degenerative Cervical Myelopathy

**DOI:** 10.3390/jcm9061828

**Published:** 2020-06-11

**Authors:** Stefania d’Avanzo, Marco Ciavarro, Luigi Pavone, Gabriele Pasqua, Francesco Ricciardi, Marcello Bartolo, Domenico Solari, Teresa Somma, Oreste de Divitiis, Paolo Cappabianca, Gualtiero Innocenzi

**Affiliations:** 1 Division of Neurosurgery, Department of Neurosciences, Reproductive and Odontostomatological Sciences, Federico II University of Naples, 80126 Naples, Italy; domenico.solari@unina.it (D.S.); teresa.somma85@gmail.com (T.S.); dedivitiis.oreste@gmail.com (O.d.D.); paolo.cappabianca@unina.it (P.C.); 2 I.R.C.C.S. Neuromed, 86077 Pozzilli, Italy; marcociavarro@gmail.com (M.C.); bioingegneria@neuromed.it (L.P.); fricciardi1@yahoo.it (F.R.); bartolonrx@gmail.com (M.B.); innocenzigualtiero@tiscali.it (G.I.); 3 Medicine and Health Science Department, University of Molise, 86100 Campobasso, Italy; ing.gabrielepasqua@gmail.com; 4 Human Neuroscience Department, Sapienza University of Rome, 00185 Rome, Italy

**Keywords:** diffusion tensor imaging (DTI), fractional anisotropy (FA), cervical MRI, degenerative cervical myelopathy (DCM), myelopathy hand

## Abstract

(1) Background: In addition to conventional magnetic resonance imaging (MRI), diffusion tensor imaging (DTI) has been investigated as a potential diagnostic and predictive tool for patients with degenerative cervical myelopathy (DCM). In this preliminary study, we evaluated the use of quantitative DTI in the clinical practice as a possible measure to correlate with upper limbs function. (2) Methods: A total of 11 patients were enrolled in this prospective observational study. Fractional anisotropy (FA) values was extracted from DTI data before and after surgery using a GE Signa 1.5 T MRI scanner. The Nine-Hole Peg Test and a digital dynamometer were used to measure dexterity and hand strength, respectively. (3) Results: We found a significant increase of FA values after surgery, in particular below the most compressed level (*p* = 0.044) as well as an improvement in postoperative dexterity and hand strength. Postoperative FA values moderately correlate with hand dexterity (r = 0.4272, R_2_ = 0.0735, *p* = 0.19 for the right hand; r = 0.2087, R_2_ = 0.2265, *p* = 0.53 for the left hand). (4) Conclusion: FA may be used as a marker of myelopathy and could represent a promising diagnostic value in patients affected by DCM. Surgical decompression can improve the clinical outcome of these patients, especially in terms of the control of finger-hand coordination and dexterity.

## 1. Introduction

Degenerative cervical myelopathy (DCM) is the most common non-traumatic spinal cord disorder in patients over 55 years old [1,2,3,4]: It is a progressive spinal cord disease characterized by degenerative changes of the bone, ligaments and intervertebral disc of cervical spine [5,6].

DCM comprises a wide set of clinical features, including neck pain, motor and sensory deficits and bladder dysfunction [7,8]. Furthermore, peculiar loss of strength and hand dexterity (the so-called “clumsy hand” or “myelopathy hand”) is observed in patients with DCM. Ono et al. [8] first reported a loss of intensity of adduction and extension in the ulnar of two or three fingers and an inability to grip and release rapidly with these fingers. Furthermore, the occurrence of “myelopathy hand” has been demonstrated to be a crucial clinical sign to achieve an early suspicion of pyramidal tract damage [9,10,11].

Conventional T2-weighted magnetic resonance imaging (T2WI) is an integral part of DCM patient evaluation. There is some evidence from a largest prospective multicenter magnetic resonance imaging (MRI) study that signal changes have some relevance in terms of its correlation with baseline and outcome. T2WI shows an increased signal intensity in the compressed part of the spinal cord; however, this abnormal MR signal has low sensitivity for structural change of the cord in cervical myelopathy and it is not predictive of neurological function before and after surgical treatment [12,13,14,15,16,17].

Diffusion tensor imaging (DTI) is an advanced imaging technique that has been proposed to assess DCM-associated demyelination and axonal damage. DTI provides also quantitative information about the white matter microarchitecture. Fractional anisotropy (FA) is a quantitative DTI parameter that measures the tendency of water to spread in a preferred direction within a group of axons and it is a function of the axonal density and integrity of white matter fibers as well as the degree of myelination [18,19,20]. Normative values of FA for healthy subjects were found to be 0.68 ± 0.05, after correcting for age and sex. Hence, a decrease of FA value highlights fiber tracts impairment. Several studies showed significant decrease of FA values at the most compressed level, but also at distant areas [21,22].

DTI values, as compared to conventional MRI, are more sensitive in the detection of DCM patients, especially in the early stage of the disease; quantitative analysis of its parameters helps in the definition of myelopathy severity and can predict the outcomes of surgical treatments [23,24,25,26].

In this study, we aimed to define the diagnostic value of quantitative DTI in patients with DCM, measuring the correlation between the FA values and hand-motor performance, i.e., dexterity and hand strength—as measured via function test batteries, thus determining its functional relevance.

## 2. Experimental Section

### 2.1. Participants

This is a prospective observational study reporting preliminary data on 11 patients (6 females and 5 males; mean age 57.64 ± 10.47 years). All subjects included in the sample were diagnosed with degenerative cervical myelopathy, as well as with myelopathic hand. The severity of cervical myelopathy was assessed using the modified Japanese Orthopedic Association (mJOA) score [27]. Each patient underwent surgery via anterior cervical discectomy and fusion (ACDF) at the I.R.C.C.S. Neuromed di Pozzilli (Isernia, Italy). A 1.5 T MRI scan from 24 to 48 h prior to the surgery and another MRI scan 3 months after the surgery were acquired for each patient. In order to evaluate the severity of the clumsy hand, the measurement of strength and hand dexterity was performed the same day of the MRI scans. Patients with cerebral palsy, rheumatoid arthritis or other spinal diseases were excluded from this study. Protocol was approved by the Ethical Committee of the I.R.C.C.S. Neuromed (Ethical Approval Code: 11/17 21-12-2017).

### 2.2. Diffusion Tensor Imaging (DTI) Acquisition and Analysis

In order to study and quantify changes in white matter structural integrity, patients underwent a cervical MRI scan with Diffusion Tensor Imaging (DTI), with a focus at the pathological segment, 24–48 h before and three months after the surgery. A GE Signa 1.5 T MRI scanner was used to acquire MRI data. The MRI protocol included Structural 2D T2-weighted images (Slice Thickness 4 mm, Repetition Time 6700 ms, Echo Time 95.9 ms, Matrix Size 320 × 224, Field of View 220 mm, Flip Angle 90°) acquired both in the axial and sagittal plane, and DTI images with 16 diffusion directions (b = 1000 s/mm^2^, 1b0, Repetition Time 10,000 ms, Echo Time 100 ms, Matrix Size 92 × 64). Image analysis was performed using the 3D Slicer software [28] and Fractional Anisotropy (FA) was extracted from DTI data [29,30]. Image processing pipeline comprised registration of anatomical T2WI with DTI images, using a 27 degrees of freedom BSpline registration. The accuracy of registration was visually assessed by a neuroradiologist (MB). Different Regions of Interest (ROIs), using the T2 images as reference, were created on a color-coded FA map in correspondence of the anatomical levels C2-C3, C3-C4, C4-C5, C5-C6 and C6-C7 to compute the aforementioned DTI parameters. The ROIs were designed by neuroradiologist and neurosurgeon (MB and GI), including both the white matter and the grey matter and excluding the cerebrospinal fluid (CSF), as described by Thurnher et al. [31].

### 2.3. Measurement of Dexterity and Hand Strength

The Nine-Hole Peg Test (NHPT) was used to assess the “digital dexterity” of the hand. Each subject performed “fine” grasping movement of nine pegs and released them in a wooden base. Once this phase was completed, the patient was instructed to remove each peg one by one, with the same hand. The test ended when all the pegs were placed inside the lid. The patients repeated the test twice for each hand and the average execution time was taken as result [32].

**Figure 1:**
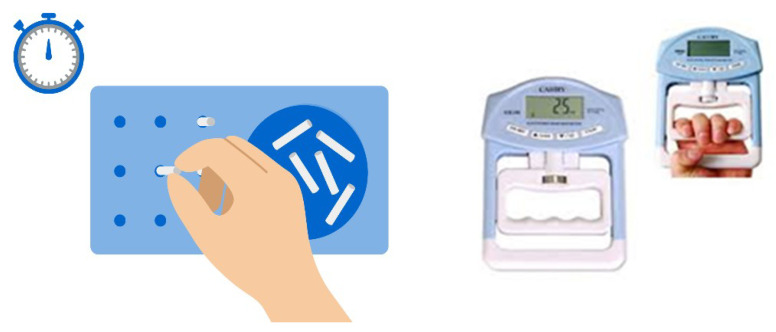
On the left: Nine-Hole Peg Test (NHPT). On the right: digital dynamometer (Camry EH101).

“Jamar” type digital dynamometer was used to measure the hand strength. The patient was instructed to sit down with the trunk in a neutral position, with the abducted shoulders aligned with each other on the frontal plane, the elbow flexed at 90°, the forearm in a neutral position, the wrist in extension between 0° and 20° and with an ulnar deviation between 0° and 15°. The dynamometer was supported by the operator’s hand to prevent possible loss of strength by the patient. Using the same setting, for the dexterity assessment the patients performed two tests and then the average value was taken as result. The first rehearsal was conducted with the dominant limb (right for all the patients) [33] (Figure 1). All these tests were performed 24–48 h before and three months after surgery.

### 2.4. Statistical Analysis

Data were analyzed using a paired *t*-test to compare the preoperative and postoperative Fractional Anisotropy values, dexterity and hand strength. A Pearson correlation analysis was then performed to assess the correlation between the postoperative FA values, strength and hand dexterity. A *p*-value < 0.05 was considered to be statistically significant.

## 3. Results

### 3.1. Baseline Characteristics

All patients underwent anterior cervical discectomy and fusion (ACDF) at 1 or 2 cervical levels. Eight patients (72.7%) showed high signal intensity (HSI) on T2WI; the mean mJOA score was 13.27 ± 2.61, therefore moderate myelopathy has been diagnosed in most patients (Table 1).

### 3.2. Diffusion Tensor Imaging (DTI)

**Figure 2:**
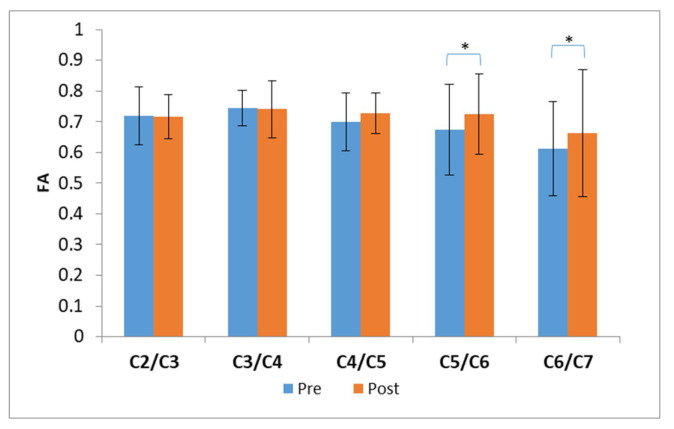
Fractional anisotropy (FA) values. Data are shown as mean values. The FA corresponding to the pre-surgery evaluation are shown in blue, whereas the FA corresponding to the post-surgery evaluation are shown in orange. Significant differences are shown with * (*p* < 0.05).

The preoperative FA values were pathological (<0.68 ± 0.05 according to [20,21]) only at C6/C7 level, mostly below the frequently compromised level (Table 2). A significant increase in FA values after surgery was found at C5/C6 and C6/C7 level (paired *t*-test, *p* = 0.005 and *p* = 0.002 respectively) (Figure 2).

We computed the FA values in correspondence of the most compressed anatomical level, which was the target of the surgery, and also in the anatomical levels immediately above and below.

**Figure 3:**
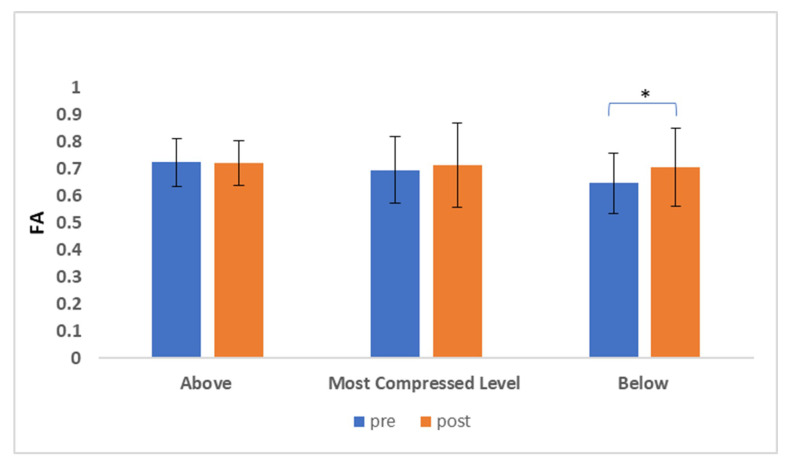
FA values of the most compressed level, the site of surgery, and the anatomical levels immediately above and below. Data are shown as mean values. The FA corresponding to the pre-surgery evaluation are shown in blue, whereas the FA corresponding to the post-surgery evaluation are shown in orange. Significant differences are shown with * (*p* < 0.05).

The preoperative FA value was pathological (Table 3) below the most compressed level; in fact, a statistically significant increase of FA postoperatively was observed at the lower level (*p* = 0.044). (Figure 3).

### 3.3. Dexterity and Hand Strength

**Figure 4:**
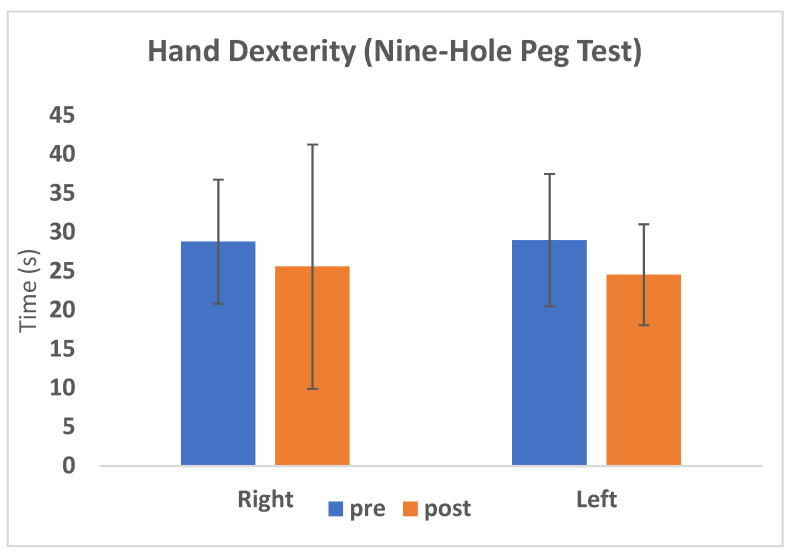
The average values of hand dexterity corresponding to the pre-surgery evaluation are shown in blue, whereas the values of hand dexterity corresponding to the post-surgery evaluation are shown in orange, distinguishing between right and left hand.

During the preoperative and postoperative evaluations, the mean values of hand “dexterity” were 28.8 and 25.6 s for the right hand, respectively, and 29 and 24.5 s for the left hand, respectively (Table 4). There was a significant improvement of hand “dexterity” (*p* = 0.002) for the left hand with a 15.4% reduction in the time needed for completing the task, while for the right hand, the improvement was not statistically significant (*p* = 0.057) (Figure 4).

**Figure 5:**
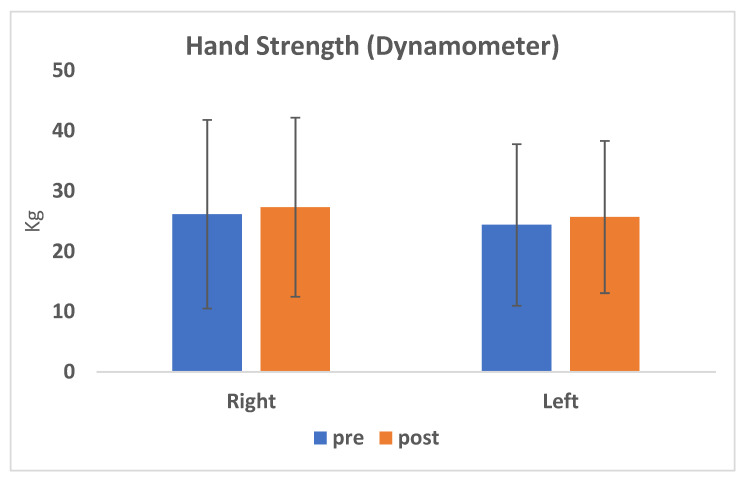
The average values of hand strength corresponding to the pre-surgery evaluation are shown in blue, whereas the values of hand strength corresponding to the post-surgery evaluation are shown in orange, distinguishing between right and left hand.

Furthermore, the mean values of right hand strength were 26.2 vs. 27.4 kg, while the mean values of left hand strength were 24.4 and 25.7 kg (Table 4); no significant improvement of the postoperative strength was found for both hands (*p* = 0.055 and 0.068) (Figure 5).

### 3.4. Correlation between the Postoperative FA Values and Strength and Hand Dexterity

**Figure 6:**
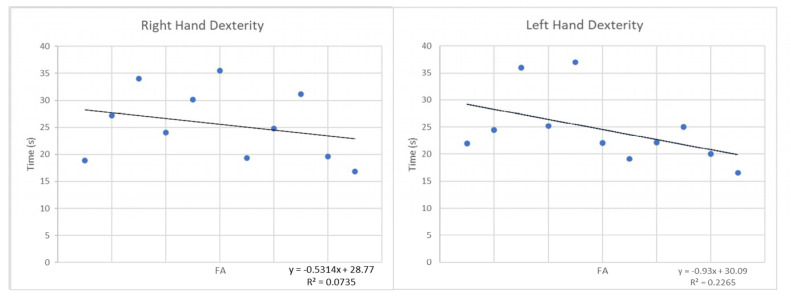
Correlation between the postoperative FA values and hand dexterity.

A weak linear correlation between the postoperative FA values measured at lower surgical level and the dexterity scores was observed; indeed, the time to perform fine grasping task was inversely proportional to the FA value (Pearson coefficient r = 0.4272, coefficient of determination R_2_ = 0.0735, *p*-value = 0.19 for the right hand; r = 0.2087, R_2_ = 0.2265, *p*-value = 0.53 for the left hand) (Figure 6).

**Figure 7:**
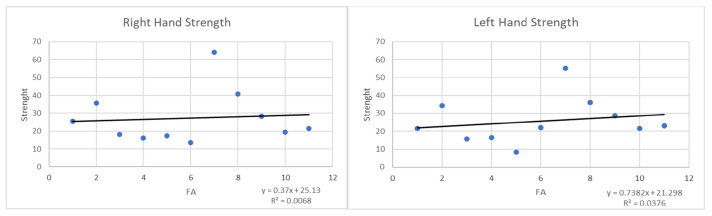
Correlation between the postoperative FA values and hand strength.

The FA values were also positively correlated with the postoperative hand strength data: There was a weak correlation between the two variables, so that the higher were the FA values, the higher was the strength of each hand (r = −0.0216, R_2_ = 0.0068, *p*-value = 0.95 for the right hand; r = 0.0035 R_2_ = 0.0376, *p*-value = 0.99 for the left hand) (Figure 7).

## 4. Discussion

Degenerative cervical myelopathy (DCM) is an insidiously progressive condition, usually showing a chronic course of clinical symptoms: impairment of gait, weakness, spasticity, clumsy hands, and sphincter disorders [1,2,3,4,5,6,7]. Conventional MR examination has played a key role in the diagnosis of cervical spondylosis, proving hypertrophy of the posterior longitudinal ligament and ligament flavum, cervical disc herniation, and cervical spinal stenosis. The compressed part of the spinal cord shows a specific high signal intensity (HSI) on T2WI. T2 HSI is often used to diagnose DCM, but this finding is not observed in each patient with clinical signs of myelopathy and its sensitivity is reported to be quite low (between 15% and 65%). Additionally, T2 HSI is generally observed only in the later stages of the disease [12,13,14,15,16,17]. A promising MR technique, diffusion tensor imaging (DTI), has been investigated for estimating the neural tissue integrity in spinal cord. As compared to conventional MR imaging, DTI parameters are more sensitive in detecting DCM, especially at the early stages [18,19,20,21,22,23,24,25,26] and they might represent a helpful tool for educating and monitoring subjects with asymptomatic spinal cord compression [34].

Unlike free water, water molecule diffusion in human is hindered by cell alignment pattern, cell membranes and other intracellular and extracellular structures showing anisotropy. Fractional anisotropy (FA) measures the tendency of water to spread in a preferred direction within a group of axons. It is a function of the axonal density and integrity of white matter fibers, as well as of their degree of myelination [35]. A decrease of FA corresponds to a damage of pyramidal tracts; in fact its value is significantly reduced in DCM patients, as compared to healthy subjects [20]. In our sample, the FA value was pathological (<0.68 ± 0.05 [21,22]) only below the most compressed level, i.e., below the segment approached surgically. It is also important to recognize that FA measures at the site of compression are sometimes difficult to obtain particularly in patients with considerable cord compression. We observed a significant increase in FA values in the postoperative course at the level just below the most compressed one, supporting that this parameter depicts a structural damage of the descending pyramidal pathways. This finding proves that DCM-associated demyelination and axonal damage afflicted both the myelopathic lesion and the distal sites over the chronic course of the disease [2].

Recent studies demonstrated a strong correlation between FA and specific clinical assessments, including mJOA score [17,18,19,20,21,22,23,24,25,26]. Shen et al. [23] showed that the mJOA score is a reasonable predictor of surgical outcome in DCM; nonetheless, a model inclusive of FA value provides superior predictive ability. Rajasekaran et al. claimed that a postoperative worsening of DTI indices is associated with a poor prognosis for neurological recovery [36]. Dong et al. [20] asserted that FA value of spinal cord was associated with postoperative recovery of spinal cord function and that DTI may play a significant role in diagnosing and predicting the development of DCM. The patients with severe DCM, who presented a higher FA value at the compressed level, were most likely to achieve a better functional recovery after decompression surgery [37]. This might identify FA as a potential positive predicting factor of postoperative outcomes: Therefore, DTI could be considered not only a complementary diagnostic analysis, but rather a crucial tool in order to identify the best candidates to surgery [38]. 

In particular, our study focused on one of the most common disorders in DCM patients: The myelopathy hand. Finger disability is a typical sign of degeneration of the corticospinal tracts and occurs only in patients with spinal cord lesions above C6-C7 level [9,10]. Doita et al. [39] showed a good correlation between the more severe cervical myelopathy and the loss of hand dexterity; Murphy et al. [40] demonstrated a strong correlation between the Nine-Hole Peg Test (NHPT) and FA values, showing that patients with moderate myelopathy performed the test in a longer time as compared to the control cases. The hand strength significantly differs between healthy subjects and myelopathic patients and its value is often influenced by age and sex.

Our patients show a more evident impairment of hand function in performing a precision grip, as assessed by the NHPT, a specific test which is regularly performed to evaluate manual dexterity in patients with multiple sclerosis and which was previously used to distinguish healthy subjects from patients with DCM. The hand “dexterity” was improved three months after surgery (11.1% for the right hand (Patient 1; 2; 4; 5; 6; 7; 10; 11), 15.4% for the left hand (Patient 1; 2; 3; 4; 5; 6; 7; 8; 10; 11)), with a moderate correlation between postoperative FA values and dexterity data; therefore, the time to carry out the test was reduced as FA values increase. The hand strength measured using a digital dynamometer showed a slight improvement at postoperative follow-up (4.4% for the right hand, 5.4% for the left hand); however, it was weakly associated with postoperative FA values.

In these patients, the damage of the corticospinal tracts determines a finger “spasticity”, which is evident when the patient is asked to reopen the hand previously forcibly closed with abnormal prolongation of voluntary de-contracting. Spasticity represents a complex clinical sign that greatly compromises the hand dexterity and the ability in performing voluntary movements in myelopathic patients [41].

Our results confirmed that FA can be claimed as a marker of myelopathy presenting both diagnostic and potential prognostic value in patient affected by DCM, as depicting the functional status of the spinal cord. Indeed, surgical decompression can improve the clinical outcomes of these patients, especially in terms of control of “fine” grasping.

Nevertheless, our study has several limitations. First, the MRI scanner used is 1.5 T, which has a good enough resolution for DTI analysis, even though 3 T scanners can have better performance. The lack of a gold standard for the diagnostic imaging of DCM and the current high standard technical requirements for diffusion weighted imaging could represent the biases of this research. Finally, another limitation of the study is the relatively small sample size of our patients.

## 5. Conclusions

The diagnosis of degenerative cervical myelopathy includes a complex clinical picture: The patient’s history, imaging and neurological status. The introduction of DTI allowed detecting spinal cord damage even at the earlier myelopathy stages, compared to the T2-weighted MR features. The combination of advanced imaging methods and diagnostic clinical tests for “Clumsy Hand” can help to accurately select the patients to be treated surgically and also to provide promising details in terms of predicting the outcomes. Further studies with larger case series and longer follow-up are needed to validate our results.

## Figures and Tables

**Table 1:** Demographic, MRI characteristics and surgical level.

Case	Gender	Surgical Level	Age	T2 Hyperintensity Signal	Most Compressed Level	mJOA Pre-op
1	F	2	57	+	C5-C6	13
2	M	2	66	+	C3-C4	12
3	F	1	75	-	C5-C6	11
4	M	2	55	-	C5-C6	9
5	F	2	56	+	C5-C6	16
6	F	1	59	+	C3-C4	12
7	M	2	38	+	C5-C6	11
8	M	1	56	+	C5-C6	14
9	M	1	67	+	C5-C6	14
10	F	2	62	-	C4-C5	17
11	F	1	43	-	C5-C6	17

F: female; M: male; mJOA: modified Japanese Orthopedic Association score.

**Table 2:** Fractional anisotropy (FA) values of the anatomical level.

FA	C2/C3	C3/C4	C4/C5	C5/C6	C6/C7
PRE	0.72	0.74	0.70	0.67	0.61
POST	0.72	0.74	0.73	0.72	0.66

**Table 3:** FA values of the most compressed level (site of surgery) and the upper and lower level. The pathological FA value (<0.68 ± 0.05 [20]) is shown in red color.

		Pre-Surgery (24–48 h)		Post-Surgery (Mean Follow-up 12 ± 2 Weeks)		
Case	Above FA	Most Compressed Level FA	Below FA	Above FA	Most Compressed Level FA	Below FA
1.	0.69	0.51	0.57	0.76	0.56	0.420
2.	0.63	0.76	0.70	0.60	0.71	0.66
3.	0.81	0.73	0.73	0.85	0.85	0.82
4.	0.70	0.79	0.72	0.71	0.84	0.82
5.	0.65	0.62	0.67	0.65	0.90	0.67
6.	0.82	0.73	0.73	0.72	0.52	0.60
7.	0.80	0.88	0.50	0.78	0.82	0.66
8.	0.63	0.59	0.63	0.72	0.59	0.89
9.	0.85	0.84	0.76	0.72	0.54	0.42
10.	0.77	0.54	0.40	0.78	0.74	0.59
11.	0.61	0.66	0.71	0.75	0.71	0.75
Mean	0.72 ± 0.08	0.69 ± 0.12	0.64 ± 0.11	0.72 ± 0.08	0.71 ± 0.15	0.70 ± 0.14

**Table 4:** Measurement of dexterity and hand strength. The percentage of improvements for NHPT and hand strength was reported in green.

	Right Hand						Left Hand					
	NHPT			Hand Strength			NHPT			Hand Strength		
	pre	post	%	pre	post	%	pre	post	%	pre	post	%
1	24.0	18.9	−21.3	23.4	25.6	9.4	26.4	22.0	−16.7	20.9	21.6	3.3
2	38.1	27.2	−28.6	31.3	35.8	14.4	33.1	24.5	−26.0	35.4	34.3	−3.1
3	32.4	34.0	5.1	15.1	18.2	20.6	39.2	36.0	−8.2	8.9	15.7	76.4
4	31.2	24.0	−23.0	18.5	16.2	−12.4	27.7	25.2	−9.0	20.1	16.5	−17.9
5	34.7	30.1	−13.3	14.1	17.4	23.4	47.4	37.0	−22.0	6.9	8.4	21.9
6	43.1	35.5	−17.6	12.0	13.5	13.0	28.8	22.1	−23.1	23.1	22.1	−4.3
7	20.0	19.3	−3.5	66.3	64.3	−3.1	21.3	19.1	−10.1	54.5	55.2	1.3
8	20.2	24.8	22.8	39.4	40.7	3.3	22.3	22.2	−0.7	34.0	36.0	5.9
9	23.1	31.2	35.1	29.3	28.4	−3.2	23.9	25.1	5.0	27.6	28.7	4.0
10	30.0	19.7	−34.5	18.0	19.5	8.3	30.5	20.0	−34.4	15.0	21.5	43.3
11	19.8	16.8	−14.9	20.9	21.5	2.9	18.1	16.5	−8.8	22.3	23.2	3.8
Mean	28.8	25.6	−11.1	26.2	27.4	4.4	29.0	24.5	−15.4	24.4	25.7	5.4
*p*-value	0.057			0.055			0.002			0.068		
